# Stability of supported aerosol-generated nanoparticles in liquid media

**DOI:** 10.1038/s41598-021-88510-2

**Published:** 2021-04-29

**Authors:** Sara M. Franzén, Magdalena Tasić, Christian B. M. Poulie, Martin H. Magnusson, Daniel Strand, Maria E. Messing

**Affiliations:** 1 NanoLund, Lund University, Box 118, 22100 Lund, Sweden; 2 Solid State Physics, Lund University, Box 118, 22100 Lund, Sweden; 3 Centre for Analysis and Synthesis, Lund University, Box 118, 22100 Lund, Sweden

**Keywords:** Nanoparticles, Synthesis and processing, Nanoparticles, Scanning electron microscopy, Surfaces, interfaces and thin films

## Abstract

The stability of nanoparticles and their supports are critical, but poorly understood, parameters for applications of such systems in liquid environments. Here we develop an approach to systematically investigate the stability of aerosol-generated nanoparticles after exposure to commonly used solvents using a combination of identical location-SEM and density/size analysis. We demonstrate that the choice of solvent needs to be carefully matched with both the particle and support materials. We show that thermal annealing significantly increases the adhesion of the particles and expands the scope of applications in aqueous media and for biological applications. The results clarify combinations of inorganic nanoparticles on oxide and semiconductor supports with solvents and environmental conditions that give sufficient stability. Combined, the presented methods should be of value in investigating the stability of nanoparticle systems after exposure to solvent and can be used for future developments of high-performing supported aerosol-generated nanoparticles for solvent-based applications.

## Introduction

Designed nanoparticles have found widespread applications in recent years—examples range from smart textiles^1^, to optical filters^2^, and sensors^3^. Several such emerging nanoparticle-based technologies, including superhydrophobic coatings^4^, additive manufacturing of embedded electronics^5^, and catalyzing the formation of semiconductor structures^6^, rely on nanoparticles generated directly in the gas phase as an aerosol. Aerosol generation is a continuous process where precursors are vaporized into gaseous species that nucleate and coalesce into stable particles when transported away by a carrier gas. The vaporization can be achieved using flames^7^, lasers^8^, electric sparks^9^, electric arcs^10^, and high-temperature furnaces^11^. Irrespective of vaporization technique, particle sizing and deposition onto virtually any type of support can then be performed with standard aerosol instruments^12,13^. Details of the aerosol generation and deposition processes are thoroughly described elsewhere^14^. Aerosol-generated particles have several advantages compared to particles generated by other methods. In particular, they are exceptionally well dispersed, contamination-free, do not require covalent surface modification, typically display very narrow size distributions and offer a high level of flexibility for multi-component particles^15–18^. These properties are attractive in contexts also where well-dispersed surface-bound nanoparticles are exposed to liquid media, for instance in biological and environmental applications^19,20^, surface-enhanced Raman spectroscopy^21^, and catalysis research^22^. Solvents are, however, particularly demanding environments: shear forces, solvent reactivity, and movement caused by evaporation can affect the particles. It has been well studied how surface-modified nanoparticles are affected in solutions^23,24^. However, these results can not be directly compared to aerosol-generated nanoparticles without any surface modifications. The stability of aerosol-generated nanoparticles and their supports is a critical, but unfortunately poorly understood, parameter to consider for the development for applications in liquid media. Recently, it has become possible to study nanoparticles in detail when immersed in a solvent by liquid-phase transmission electron microscopy to, e.g., visualize nanoparticle movements^25^. This technique is not, however, applicable for surface-bound nanoparticles due to the lack of transmission through the support.

**Figure 1:**
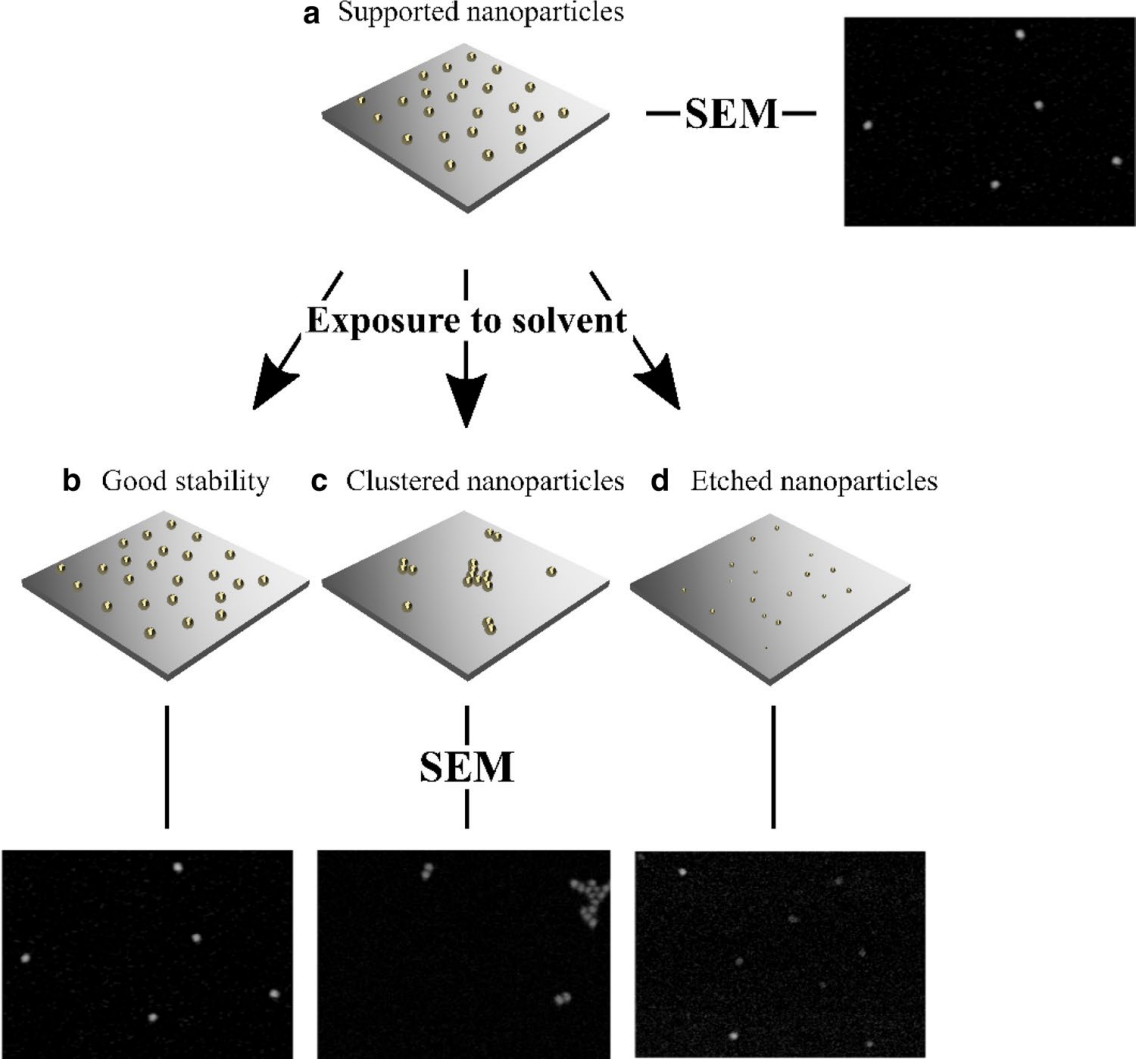
Schematics and SEM images showing the supported nanoparticles (**a**) before treatment, as well as the possible outcomes when exposed to solvent under varying conditions; (**b**) good stability; (**c**) clustered nanoparticles due to nanoparticle movement; and (**d**) etched nanoparticles.

Here we present a systematic approach, using scanning electron microscopy (SEM), to evaluate the integrity of supported aerosol-generated gold and palladium nanoparticles after exposure to various solvents. Particle movement on the surface is evaluated using identical location-SEM (IL-SEM)^26^ and etching of the particles and support surface by measuring the alterations of particle size distributions, particle density, and surface roughness (Fig. 1). Samples are compared before and after exposure to protic and aprotic solvents, as well as aqueous solutions, at varying temperatures and pH ranges. The result is a map of stability domains that can guide and expedite the design of supported gold and palladium nanoparticle systems for solvent-based applications that require well-dispersed support-bound nanoparticles.

## Results and discussion

### Design and fabrication of supported nanoparticle samples

At the outset we devised an experimental suite consisting of gold nanoparticles deposited on three different crystalline supports: gallium phosphide, silicon, and alumina. We also investigated palladium nanoparticles deposited on silicon. Silicon and gallium phosphide are commonly employed semiconductor materials for optical and optoelectrical applications^27,28^, and gallium phosphide nanostructures have also been used in a series of biological studies^29,30^, as well as photoassisted electrolysis of water^31,32^. Both the gallium phosphide and silicon supports had a passivating amorphous oxide layer on the surface. The gold nanoparticles were produced as an aerosol in a high-temperature furnace as described by Magnusson et al.^33^. The palladium nanoparticles were produced by spark ablation following the method of Meuller et al.^34^.

**Figure 2:**
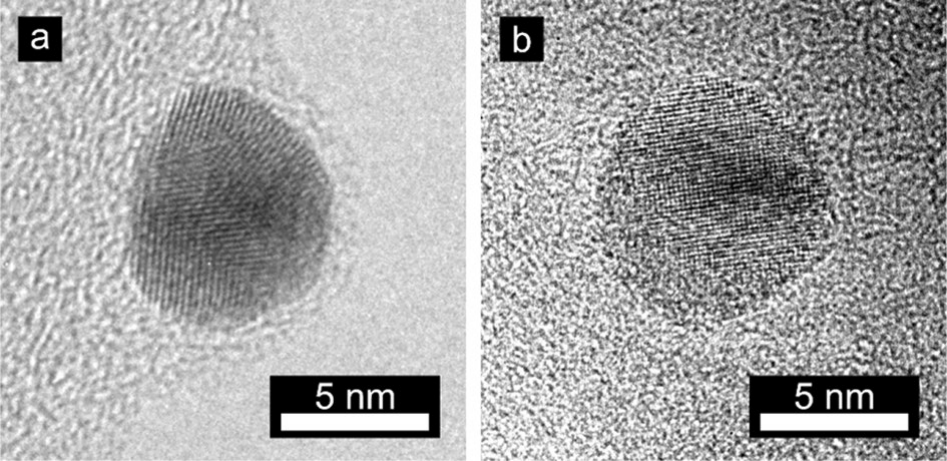
HRTEM image of aerosol-generated nano-particles: (**a**) gold nanoparticle; (**b**) palladium nanoparticle.

The resulting particles were deposited onto the respective supports with the aid of an electric field, using an electrostatic precipitator (ESP)^35^. In an ESP, the supports are positioned on a metal plate kept at a high constant electrical potential, forcing the charged particles to deviate from the gas stream and deposit onto the support with 100% efficiency^36^. The deposited nanoparticles were monodisperse in size (± 15% of the average diameter) and evenly distributed across the surface, as illustrated in Fig. 1 a; the morphology and crystallinity of typical particles are shown in the high resolution transmission electron microscopy (HRTEM) images in Fig. 2.

To strengthen the nanoparticles’ adhesion to the support, some samples were also subjected to thermal annealing (600 °C for 5 min). Because of their small size, the nanoparticles will partially melt at this temperature and create a close to hemispherical shape with an increased interface towards the support. All supports were imaged with SEM after nanoparticle deposition or after thermal annealing.

### Evaluation of stability following exposure to liquid media

To evaluate the stability of the nanoparticle supports in various solvents, the supports were immersed into the respective solvent for 24 h. The surface of the support was washed with MeOH and dried under reduced pressure (see the experimental section for details). Immersion in and removal from MeOH was shown not to cause alternations, see Table 1, Table 2 and Figure S2.

**Table 1:**
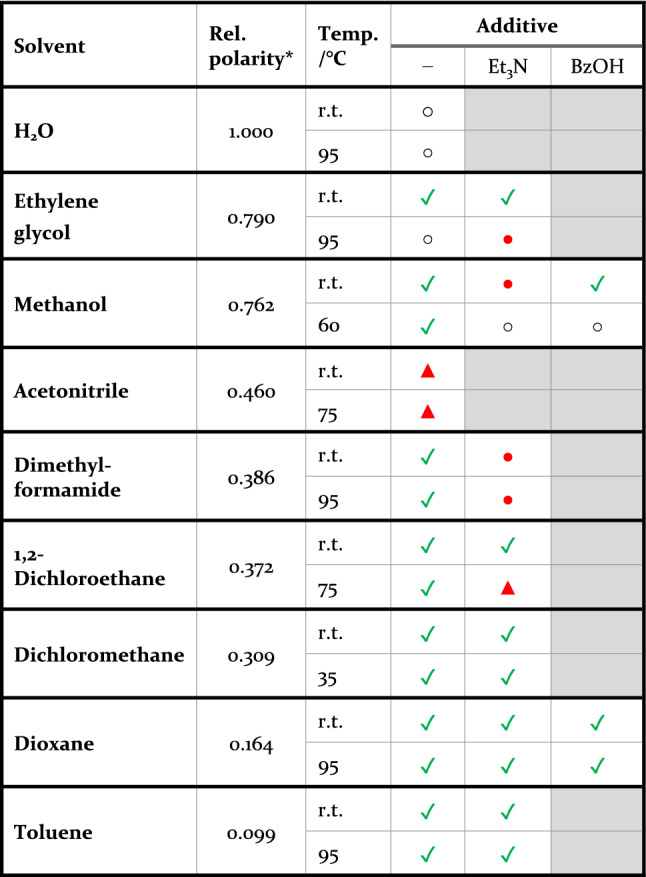
Screening of the stability of AuNP/Si supports in different solvents, with and without the addition of base/acid^a,b^.

^a^General procedure: A nanoparticle support was immersed into 1 mL solvent or 1 mL solvent and 0.05 mM triethylamine (Et_3_N) or 1 mL solvent and 0.05 mM benzoic acid (BzOH) for 24 h at room temperature or slightly below the boiling temperature of the respective solvent or to a maximum of 95 °C.

^b^Qualitative assessment:  —good stability, no movement or etching of nanoparticles; ○—minor movement of nanoparticles;  —major movement of nanoparticles;  —etched nanoparticles; grey—not tested. Each entry is based on reference images with a total area of 18 μm^2^ (~ 360 nanoparticles) per sample and at least two independent experiments.

*Taken from reference^42^.

**Table 2:**
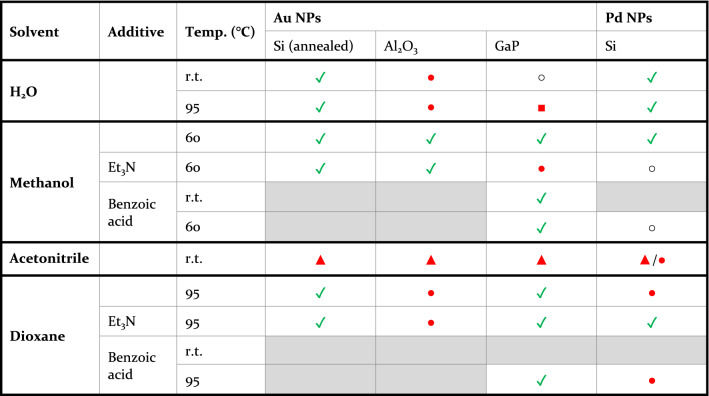
Screening of the stability of supported nanoparticles in a selection of solvents when changing the material of the nanoparticles or the support. In selected cases with the addition of base/acid^a,b^.

^a^General experimental: Nanoparticle supports immersed into 1 mL solvent or 1 mL solvent and 0.05 mM Et_3_N or 1 mL solvent and 0.05 mM BzOH for 24 h at room temperature or slightly below the boiling temperature of the solvent (maximum 95 °C).

^b^  —good stability; ○—minor movement of nanoparticles;  —major movement of nanoparticles;  —etched nanoparticles;  —etched surface; grey—not tested. Each entry is based on reference images with a total area of 18 μm^2^ (~ 360 nanoparticles) per support and at least two independent experiments.

The movement of individual nanoparticles on the surface was then evaluated using IL-SEM^26^. In brief, the crystalline support was marked prior to particle deposition and scanning electron micrographs were acquired of the same position on the sample before and after treatment in solution, referred to as reference areas. Using this method, very small lateral changes (< 10 nm) of nanoparticles and addition or removal of individual nanoparticles could be identified. In addition to the reference areas, scanning electron micrographs were acquired outside of the reference areas in order to evaluate general changes to the supported nanoparticles, such as clustering and change in nanoparticle density.

**Figure 3:**
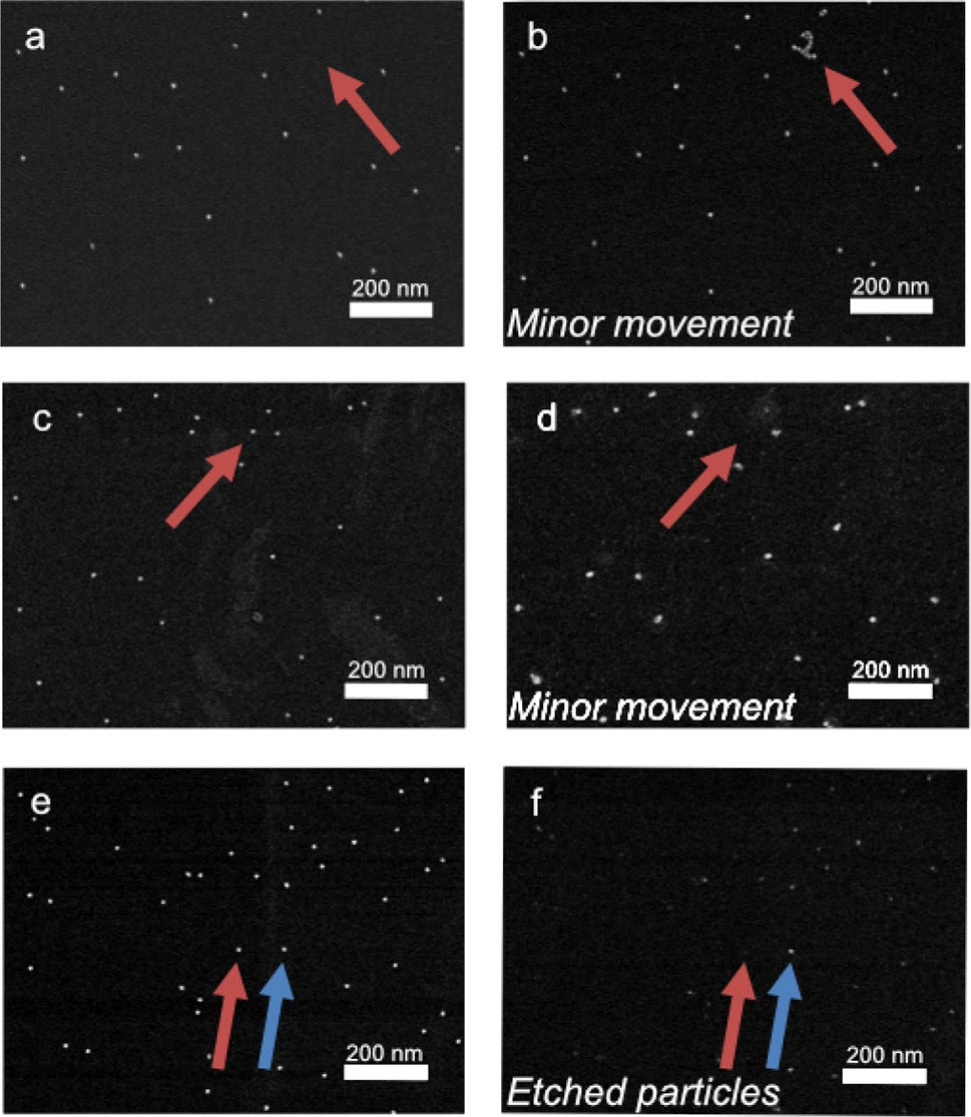
Representative IL**-**SEM images of AuNP/Si supports (**a**) before and (**b**) after treatment in room temperature H_2_O, (**c**) before and (**d**) after treatment in 95 °C H_2_O and (**e**) before and (**f**) after treatment in room temperature acetonitrile. The arrows mark examples of changes in the images before and after exposure to the solvent. The presence of additional nanoparticles in (**b**) and the absence of a few nanoparticles in (**d**) indicate minor movement of the nanoparticles. The decreased size of the particles in (**f**) compared to in (**e**) indicates that the nanoparticle has been etched.

Movement of nanoparticles was identified by the following markers: (i) nanoparticle linking/clustering (Fig. 1 c), evaluated outside the reference areas, (ii) change in nanoparticle density, evaluated outside the reference areas (> 30% loss indicates major movement), and/or (iii) movement of particles, evaluated within the reference areas (movement of 5–19 nanoparticles indicates minor movement, ≥ 20 indicates major movement). Representative examples of images before and after solution treatment are shown in Fig. 3. Etched nanoparticles were identified by measuring the size distributions of the particles before and after treatment, see the Supplementary Information, Figure S5. Etching of the support was identified by roughening of the atomically smooth crystalline surface. Using these criteria, the stability of gold nanoparticles on silicon supports (AuNP/Si) was first evaluated in a comprehensive screening that spanned both varying solvents and conditions. The results are summarized in Table 1 (for measurement data, see Table S1 in the Supplementary Information).

Gold nanoparticles supported by silicon were found to be stable in most solvents, albeit with some notable exceptions. After exposure to H_2_O at room temperature, additional nanoparticles were found in the reference areas (Fig. 3 a, b) which is a clear sign of particles migrating on the surface. When exposed to H_2_O at 95 °C, the silicon surface was also slightly roughened and the particle size had increased (Fig. 3 c, d). A possible explanation for this observation is overgrowth of the gold particles with silicon^37^. To investigate the stability of the samples also in cell growth media, we evaluated the effect of immersion in phosphate-buffered saline solution (PBS buffer). Unfortunately, large amounts of salt residues remained on the surface even after washing and drying, which prevented imaging of the nanoparticles, and no conclusive evaluation on stability could be made (Supplementary Figure S1a).

With respect to nanoparticle etching, the supported nanoparticles were found to be resilient to all protic and most aprotic organic solvents tested. The exception was the aprotic and coordinating solvent acetonitrile (MeCN), that has a well-known interaction with gold^38,39^, that gave significant etching of the nanoparticles. In this solvent, the particles were partially etched (reduced in size) after 24 h, both at room temperature (Fig. 3 f) and at an elevated temperature (75 °C) (Supplementary Figure S2). The support was unaffected under the same conditions.

Since many applications are dependent on specific pH ranges, we also investigated the stability under acidic and basic conditions in both aqueous and organic mixtures. Inorganic bases like potassium carbonate and sodium hydroxide resulted in a severe etching of both the surface and the particles (Supplementary Figure S1d). In contrast, triethylamine (Et_3_N), a soluble organic base, was well tolerated and the supports unaffected in the non-polar solvents dichloromethane, dioxane, and toluene also at elevated temperatures (35 °C, 95 °C, and 95 °C, respectively, Table 1). An exception is that triethylamine in methanol or dimethylformamide resulted in significant movement of particles on the support surface. For triethylamine in methanol, more movement was observed at room temperature than at increased temperature. The differences were, however, small, and likely attributed to experimental variations (for experimental data on nanoparticle movements, see Supplementary Information Table S1). Another limitation is that in 1,2-dichloroethane/trimethylamine at elevated temperature (75 °C), the particles were etched. To probe the acidic part of the pH spectrum, we employed benzoic acid as a soluble organic acid and evaluated stability in methanol and dioxane. The supported nanoparticles were stable in dioxane and showed relatively good stability also in methanol, indicating a higher tolerance to acidic than to basic conditions.

There were no clear correlations between the polarity of the solvent and the nanoparticle stability. However, the silicon-supported gold nanoparticles showed good stability in all of the tested non-polar solvents 1,2-dichloroethane, dichloromethane, dioxane, and toluene, and in most cases, also with the addition of a base or an acid.

**Figure 4:**
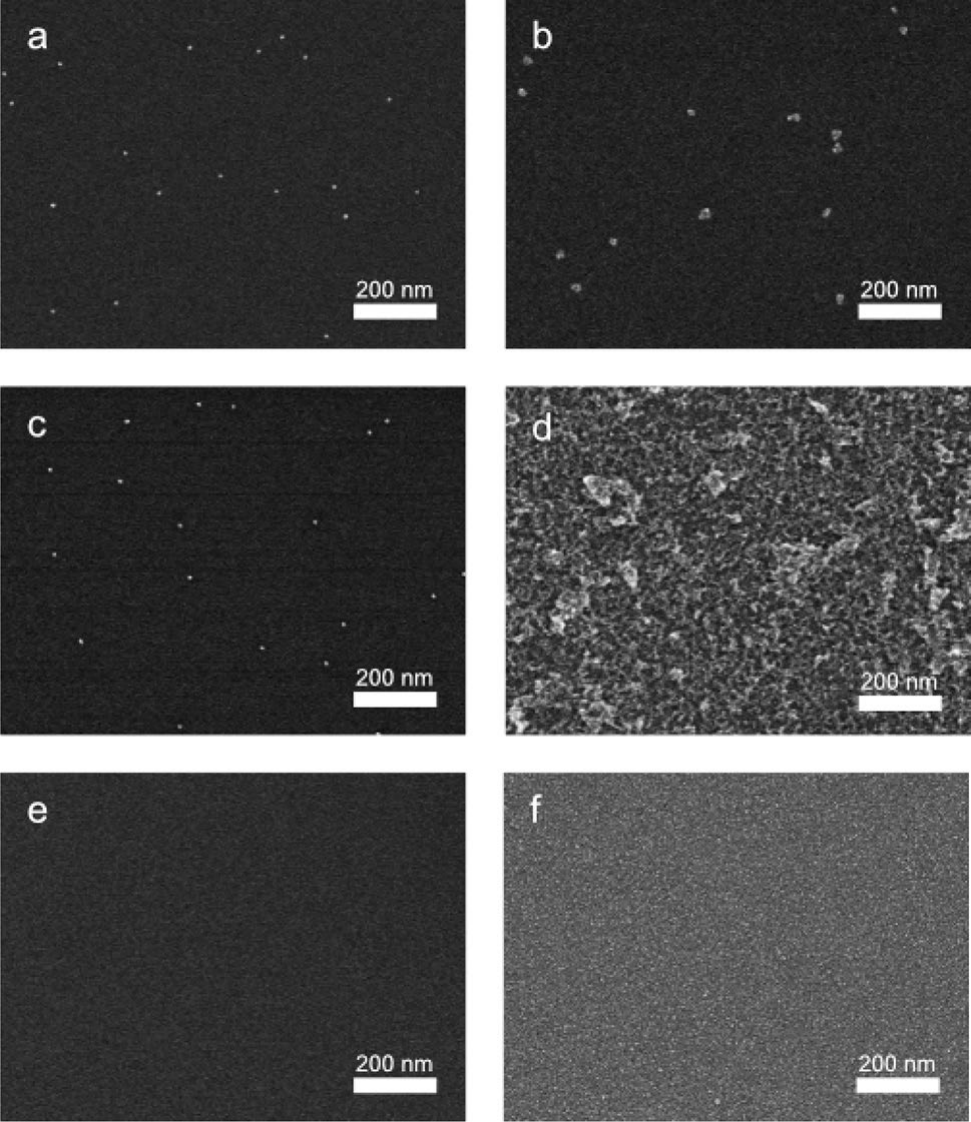
PdNP/Si chip (**a**) before and (**b**) after treatment in 95 °C dioxane. Clustering of nanoparticles into small, compact agglomerates. AuNP/GaP chip (**c**) before and (**d**) after treatment at 95 °C in H_2_O. Significant etching of the surface, resulting in the complete removal of the nanoparticles from the support. GaP chip without nanoparticles (**e**) before and (**f**) after treatment at 95 °C in H_2_O.

With a validated method and a clarified picture of the stability of gold nanoparticles on silicon at hand, we continued to investigate more combinations of support materials and nanoparticles (Table 2). For this study, we employed a select set of solvents commonly used in chemistry applications and based the selection on the results obtained for the gold/silicon system. The supported nanoparticles used in this screening were gold on gallium phosphide and alumina and palladium on silicon. Also included in this screening were gold nanoparticles on silicon, which had undergone post-deposition annealing. The results are summarized in Table 2. An interesting outcome is that nanoparticles of gold and palladium showed quite different stability in some cases. Palladium particles migrated and clustered considerably on the surface when exposed to dioxane, a solvent in which gold nanoparticles on silicon showed good stability. The micrographs also showed that the palladium nanoparticles, which remained static on the surface, were severely deformed, i.e., larger and non-spherical, in this solvent (Fig. 4 a, b). Surprisingly, the addition of triethylamine to dioxane increased the stability of palladium nanoparticles on silicon, whereas acidic, or even neutral, conditions were less well tolerated. Given this contrasting behavior, it is also worth noting that palladium nanoparticles showed a good adhesion to the silicon surface in H_2_O, a solvent that caused significant movement of gold nanoparticles. With respect to the support material, supports composed of nanoparticles on silicon and alumina showed very similar stability. On the other hand, the gallium phosphide support was unstable in water, and especially at elevated temperature (95 °C), where severe corrosion was seen (Fig. 4 c, d). Since it has been previously reported that pure GaP is stable in water^31,32^, it was suspected that the gold particles were necessary for the corrosion. Thus, plain GaP supports were tested in the same conditions, which resulted in some, although not as severe, corrosion of the surface (Fig. 4 e, f). The severe corrosion of the GaP surface with supported gold nanoparticles, compared to that of the bare surface, indicate that the gold nanoparticles are accelerating the decomposition of the surface. This result is perhaps not completely surprising, given that gold nanoparticles are extensively used to catalyze the growth of gallium phosphide nanostructures^40^.

Finally, we also investigated the stability of supports with thermally annealed gold nanoparticles. As expected, thermal annealing significantly improved the stability of the particles and these remained static even in solvents where non-annealed particles were not stable. As expected, thermal annealing did not increase the resilience of gold nanoparticles to acetonitrile.

## Conclusions

The obtained data support several conclusions. It is clear that commonly used solvents and additives can impact aerosol-generated particles and the support surfaces they are deposited on. Given the prevalence of both gold and palladium nanoparticles in heterogeneous catalysis, the results herein re-emphasize that stability and metal leaching are critical considerations in catalyst design and performance. Moreover, nanostructured gallium phosphide has been used in a number of in vitro cell-based studies, and it is clear that stability is a factor that must be carefully considered in such experiments. The corrosion of gallium phosphide surfaces, when exposed to water at elevated temperatures, underscore the importance of confirming the integrity of the nanostructures. Not least, in light of the potential impact of leached toxic elements on, for instance, cell assay results. Finally, our results show that the combination of particle and support material needs to be carefully chosen to withstand specific liquid environments.

In summary, we have further developed and applied a microscopy-based method to investigate the stability of supported nanoparticles in various solvents and conditions. Influence on the supports are characterized, both in terms of adhesion of the nanoparticles to the surface (movement upon solvent exposure) and etching of the nanoparticles and/or the support.

The stability of the nanoparticle supports was found to be strongly dependent on the solvent in which it is immersed. For each of the nanoparticle system studied, we were, however, able to identify both protic and aprotic solvent conditions where both the particle and support were stable. The addition of an organic acid was well tolerated and did not decrease stability in any tested solvents in which the nanoparticle supports were stable. On the other hand, basic additives led to weaker adhesion in some solvents, and in the case of heated dichloroethane, even to etching. Thermal annealing of the gold/silicon supports does have a positive effect on the adhesion of the nanoparticles increasing their resilience to H_2_O, even up to 95 °C. This study clearly shows that aerosol-generated nanoparticles are suitable for solvent-based applications contingent on a careful choice of support. The development of such systems applications are currently under way in our laboratories and will be reported in due course.

## Experimental/method

### Production of supported nanoparticle samples

For this study metal nanoparticles deposited onto planar, crystalline supports were used, which further on will be referred to as nanoparticle chips. The three different supports studied were Si (100), GaP (111)B, and Si (100) with a 10 nm layer of Al_2_O_3_, grown on the silicon by atomic layer deposition (ALD) using the tool Savannah S100 which cycles pulses of H_2_O and trimethylaluminum.

The palladium nanoparticles were produced by an aerosol method called spark discharge generation, which is described by Messing et al.^41^. In this method a metallic vapor is formed by spark discharges between two electrodes which are composed of the material of the intended nanoparticles. The metallic vapor nucleates to form primary particles which coalesce to form larger nanoparticles. The particles are then sintered in a tube furnace to form spherical and crystalline particles, followed by size selection with a differential mobility analyzer (DMA)^33^ and deposition by electrostatic precipitation^36^. For production and deposition of gold nanoparticles, a similar method was used, called evaporation/condensation generation^11^, where the metallic vapor is produced by heating a bulk piece of gold in a high-temperature furnace at about 1500 °C. Upon cooling, the gold vapor forms particles that are sintered and size selected in a similar set-up as for the spark discharge generator. The aerosol nanoparticles are size selected (DMA set to 10 nm) and deposited onto the support in an electrostatic precipitator (ESP), which focuses the nanoparticles onto the support by electrostatic forces.

In order to anneal the nanoparticle supports, they were heated in a rapid thermal processing (RTP) system, RTP-1200-100 from UniTemp GmbH, at 600 °C under nitrogen for 5 min.

### Exposure of samples to solvent

For organic solvents, air and water free ZerO_2_ from Sigma-Aldrich was used. For water, Milli-Q water was used. Where indicated, 0.05 mM Et_3_N (ZerO_2_) or benzoic acid was added.

#### Procedure

Supports were placed in a glass vial with nanoparticles facing upwards. To the vial was added 1.0 mL of the respective solvent or solution and the vial was sealed with a screw cap for 24 h. Where indicated, the vial was heated to the appropriate temperature (in the range of room temperature to 95 ºC) for 24 h. The vial was then cooled down to ambient temperature and the solvent was removed via a glass pipette. Nanoparticle supports exposed to high boiling point solvents were washed with 2 × 1 mL MeOH. Any remaining solvent was removed from the chips under reduced pressure (approx. 100 mbar), until dryness.

### TEM analysis

Nanoparticles generated by the aerosol methods of evaporation/condensation, both by the aid of a high-temperature furnace and by spark discharges, were deposited onto lacey carbon Cu TEM grids. A high-resolution transmission electron microscope (HRTEM, JEOL 3000F) operated at 300 kV and equipped with a field emission gun and an X-ray energy dispersive spectrometer was used for investigations of nanoparticle morphology and confirmation of nanoparticle composition. HRTEM images of aerosol-generated gold and palladium nanoparticles are shown in Fig. 2.

### Identical location-SEM analysis

This study was performed by imaging nanoparticle deposited onto a planar support before and after treatment using a Hitachi SU8010 Cold Field Emission Scanning Electron Microscope (SEM). The micrographs were acquired in reference areas identified by marks from a diamond pen; the markings were here used as unique reference points, but the markings themselves were not included in the images. The images were used for identical location analysis after treatment of the chips in order to track small changes to individual nanoparticles, and the total area that was imaged for each sample was 18 µm^2^, containing about 360 nanoparticles.

### Density/size analysis

Since exposure to the electron beam may sinter the nanoparticles, it was necessary to also analyze areas that had not previously been imaged in the SEM, i.e. to analyze images acquired outside of the reference areas. The SEM images were acquired of a total area of 22.5 µm^2^, containing about 450 nanoparticles, and analyzed using the software ImageJ and the built-in macro “Analyze particles”.

### Classification

From the IL-SEM analysis it was considered to have been *major movement* of nanoparticles if the number of changes in the reference areas (removal or movement of nanoparticles) was at least 20 nanoparticles and it was considered to have been *minor movement* of the nanoparticles if the number of changes in the reference area was 5–19 nanoparticles, i.e., about 1–5% of the nanoparticles. Another indicator of *minor movements* was the presence of additional particles in the reference areas. A large number of additional particles in the reference area was not, however, an indication of *major movement* of the nanoparticles since it could be a result of the presence of a large cluster of particles and were not representative for the nanoparticle movement in general. From the density/size analysis it was considered *major movement* if the overall number density of nanoparticles outside the reference areas had decreased by more than 30%. It was considered to be at least *minor movement* if there were linked/clustered nanoparticles.

If major movement was indicated by any of the described measures the combination was classified as *major movement*, even if another descriptor indicated only minor movement or even good stability. Similarly, if minor movement was indicated by any of the described measures the combination was classified as *minor movement*. *Etched nanoparticles* were identified by a significant change to their apparent size. *Etched surface* was identified by significant contrast shifts in the SEM images over the surface of the support.

## Supplementary Information

Supplementary Information

## Data Availability

The datasets generated during the current study are available from the corresponding authors on reasonable request.
