# Supplementary material for: Stability of supported aerosol-generated nanoparticles in liquid media
Source: Sci Rep. 2021 Apr 29;11:9276. doi: 10.1038/s41598-021-88510-2 (PMC8085219; doi:10.1038/s41598-021-88510-2)
Supplement: Supplementary file 1 — Supplementary Information [file 41598_2021_88510_MOESM1_ESM.pdf]

Stability of supported aerosol-generated nanoparticles in liquid media

Supplementary information

Sara M. Franzén, Magdalena Tasić, Christian B. M. Poulie, Martin H. Magnusson, Daniel Strand and Maria E. Messing

A. Representative SEM images acquired outside of reference areas

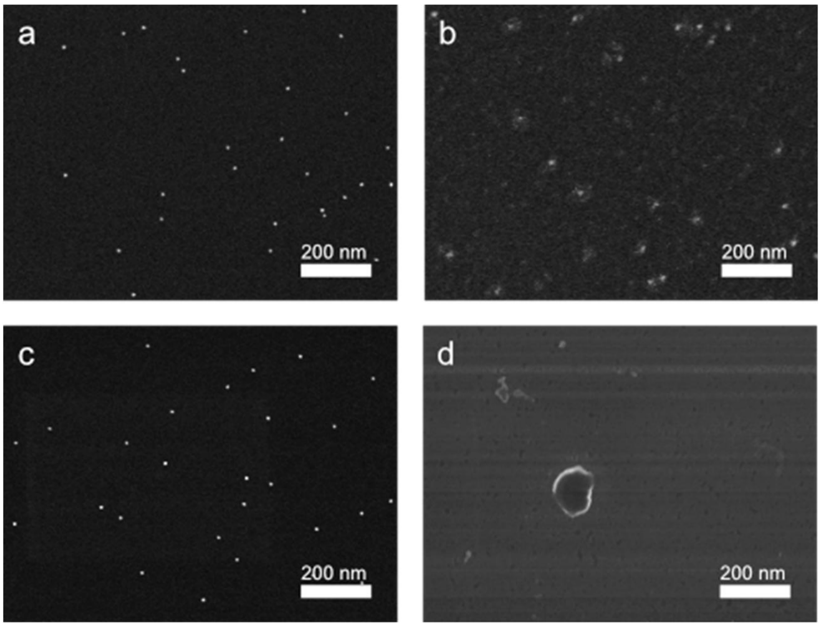

Figure S1. AuNP/Si (a) before and (b) after treatment in PBS buffer at 37 °C. AuNP/Si (c) before and (d) after treatment in K_2CO_3/MeOH at room temperature.

B. Representative IL-SEM images

In this section representative SEM images acquired within the reference areas (IL-SEM images) are shown.

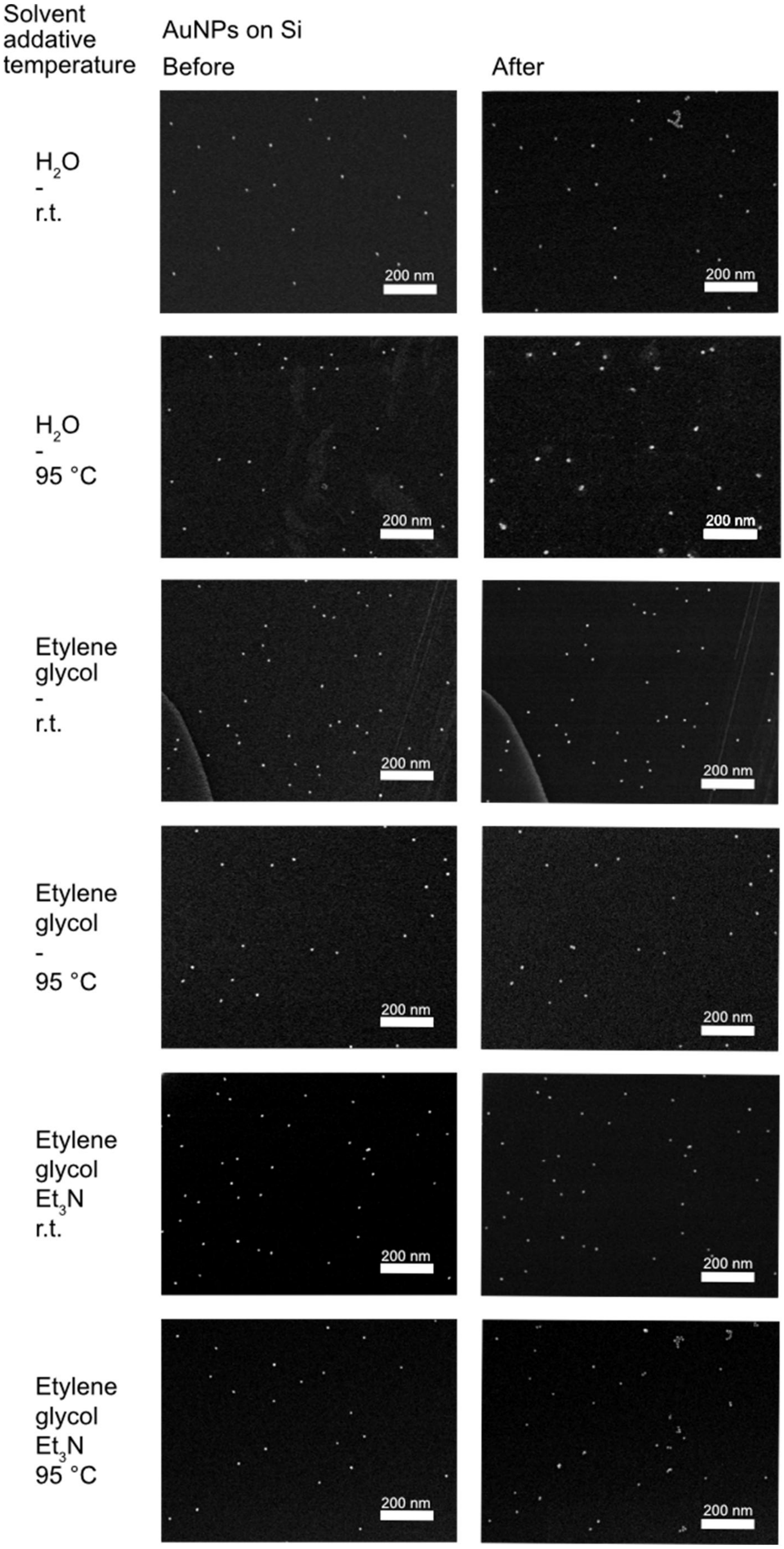

Solvent
addative
temperature
AuNPs on Si
Before
After
H2O
-
r.t.
H2O
-
95 °C
Etylene
glycol
-
r.t.
Etylene
glycol
-
95 °C
Etylene
glycol
Et3N
r.t.
Etylene
glycol
Et3N
95 °C
200 nm

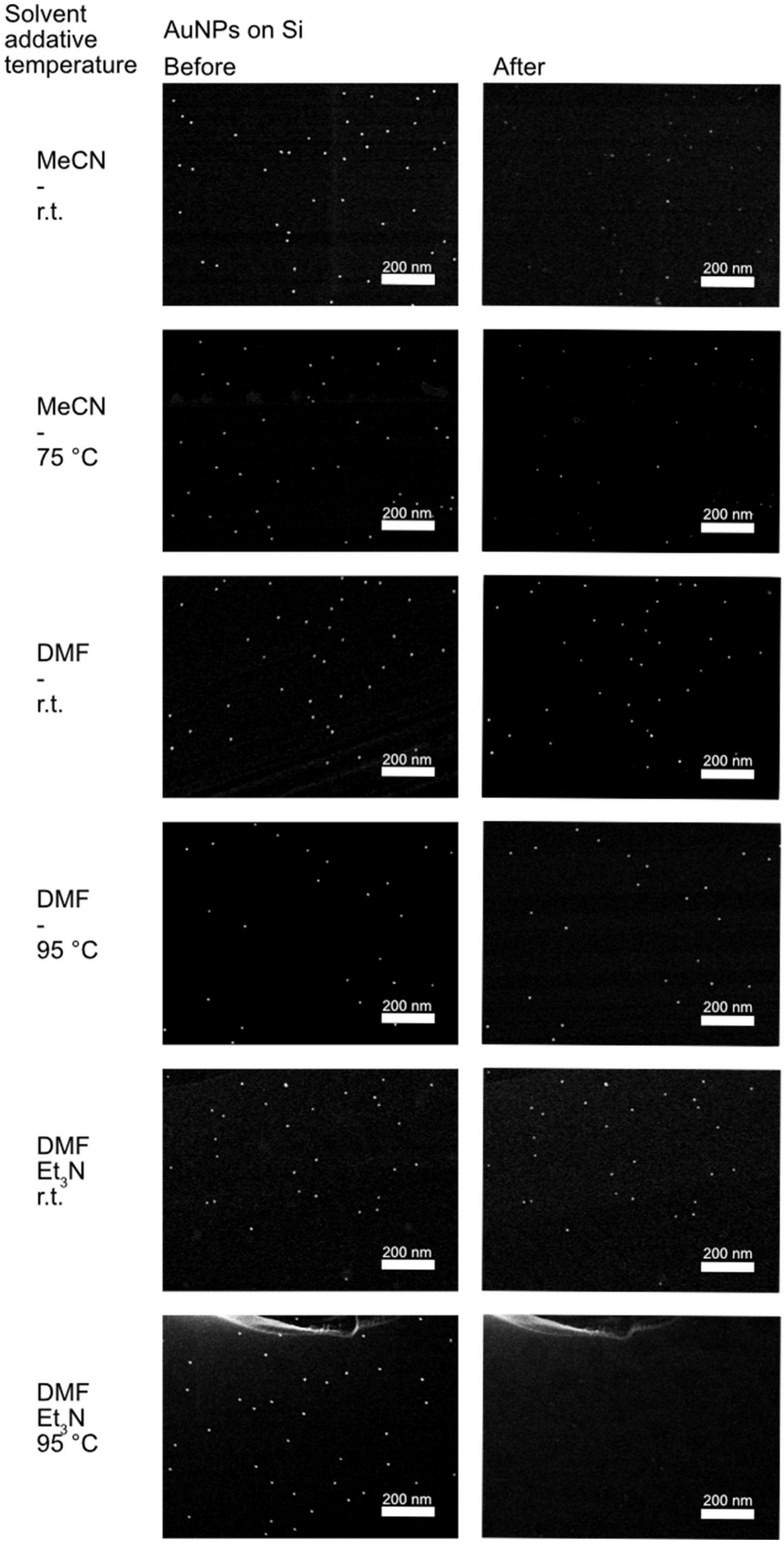
Solvent
addative
temperature
AuNPs on Si
Before
After
MeCN
-
r.t.
MeCN
-
75 °C
DMF
-
r.t.
DMF
-
95 °C
DMF
Et3N
r.t.
DMF
Et3N
95 °C
200 nm

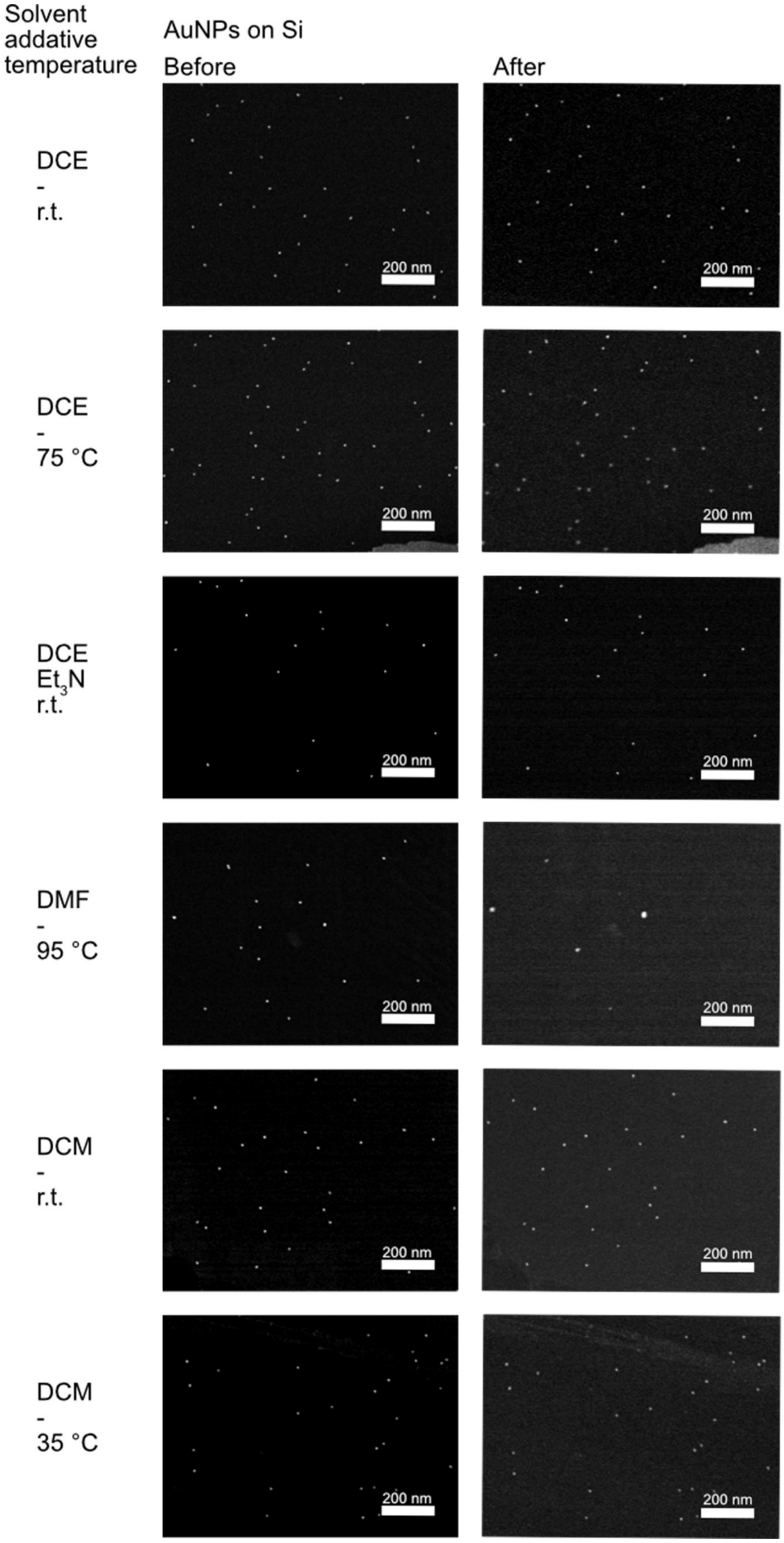
Solvent
addative
temperature
AuNPs on Si
Before
After
DCE
-
r.t.
DCE
-
75 °C
DCE
Et_3N
r.t.
DMF
-
95 °C
DCM
-
r.t.
DCM
-
35 °C
200 nm

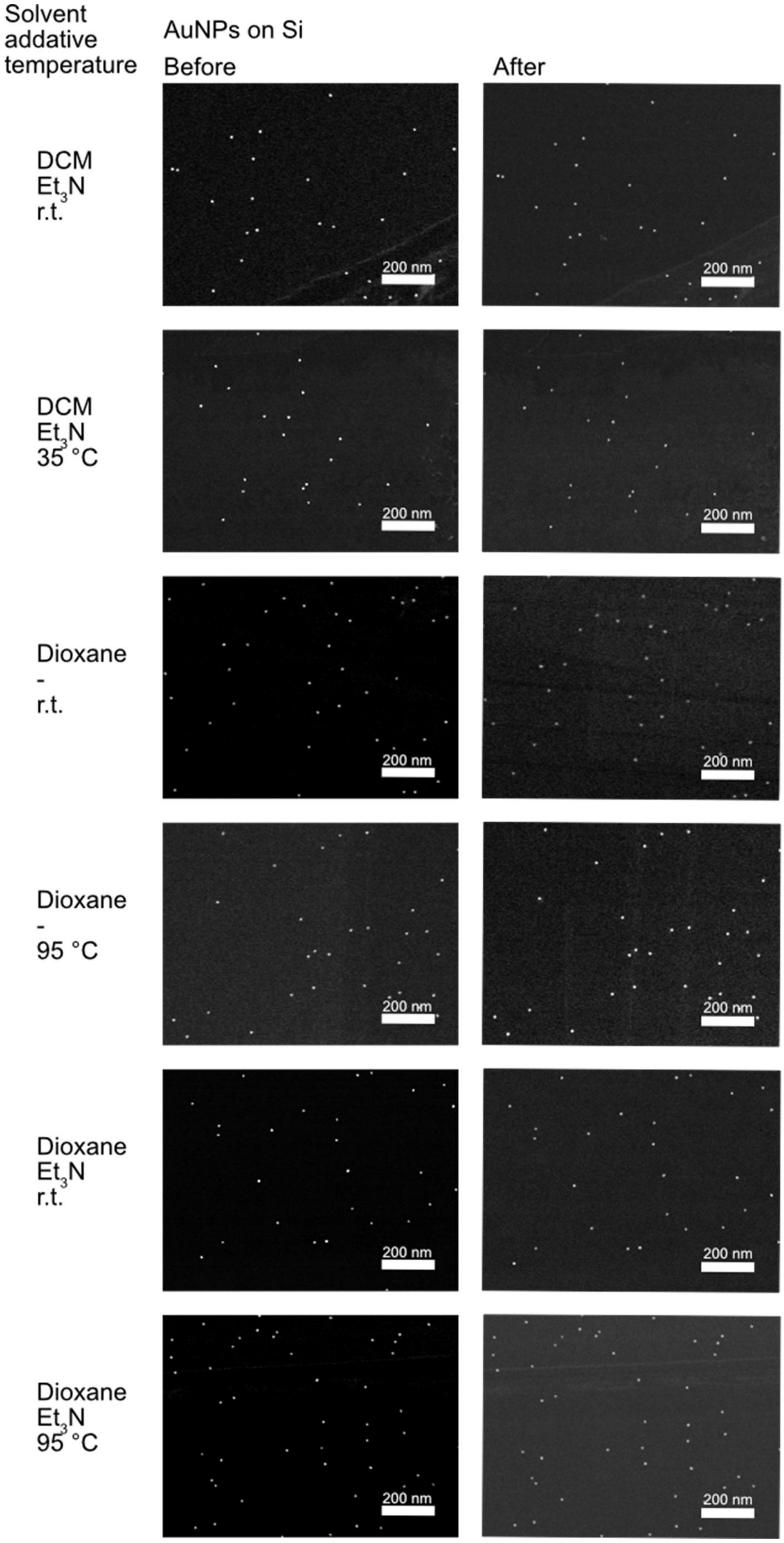
Solvent
addative
temperature
AuNPs on Si
Before
After
DCM
Et_3N
r.t.
DCM
Et_3N
35 °C
Dioxane
-
r.t.
Dioxane
-
95 °C
Dioxane
Et_3N
r.t.
Dioxane
Et_3N
95 °C
200 nm

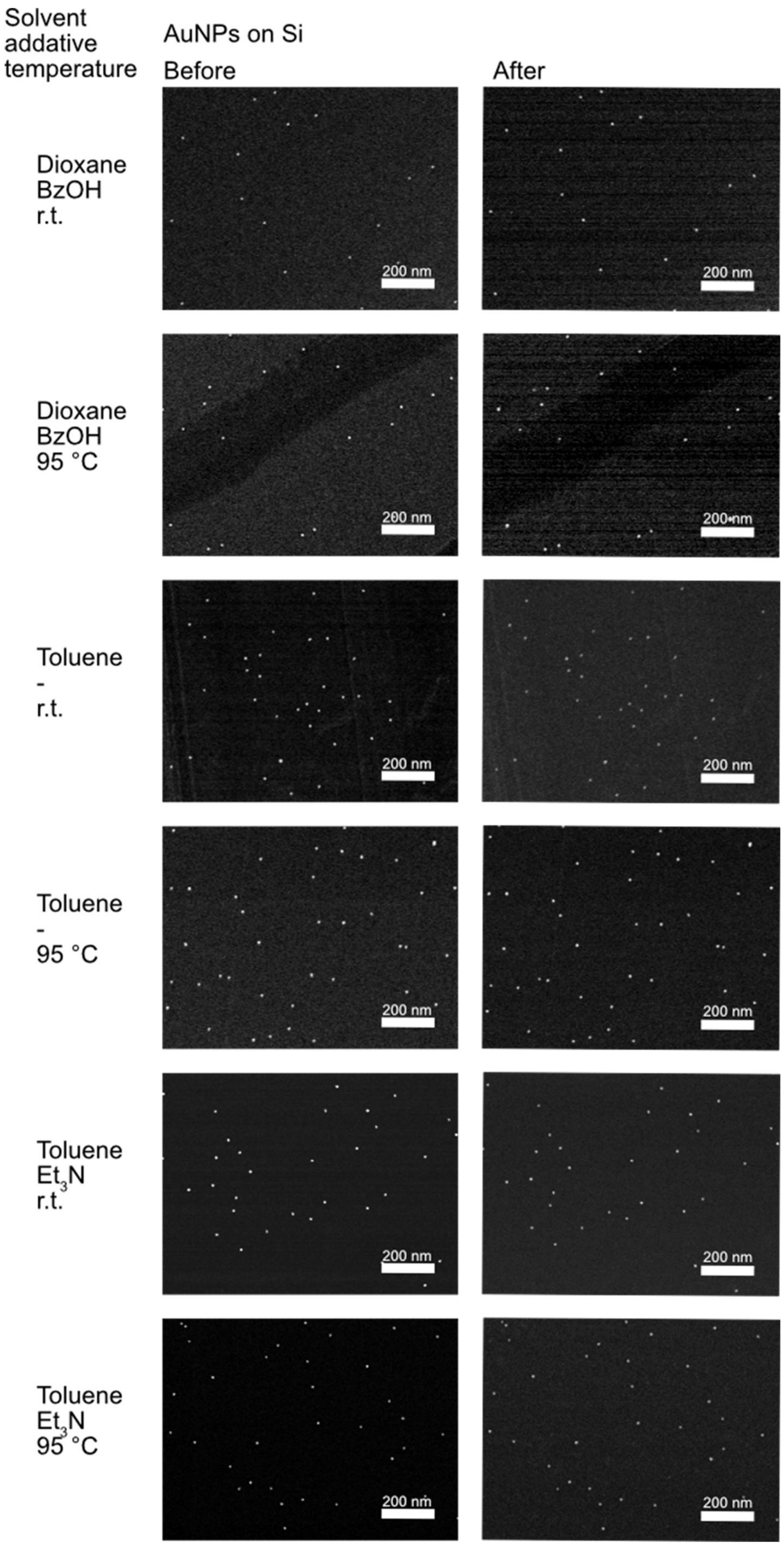

Solvent
addative
temperature
AuNPs on Si
Before
After
Dioxane
BzOH
r.t.
Dioxane
BzOH
95 °C
Toluene
-
r.t.
Toluene
-
95 °C
Toluene
Et3N
r.t.
Toluene
Et3N
95 °C
200 nm

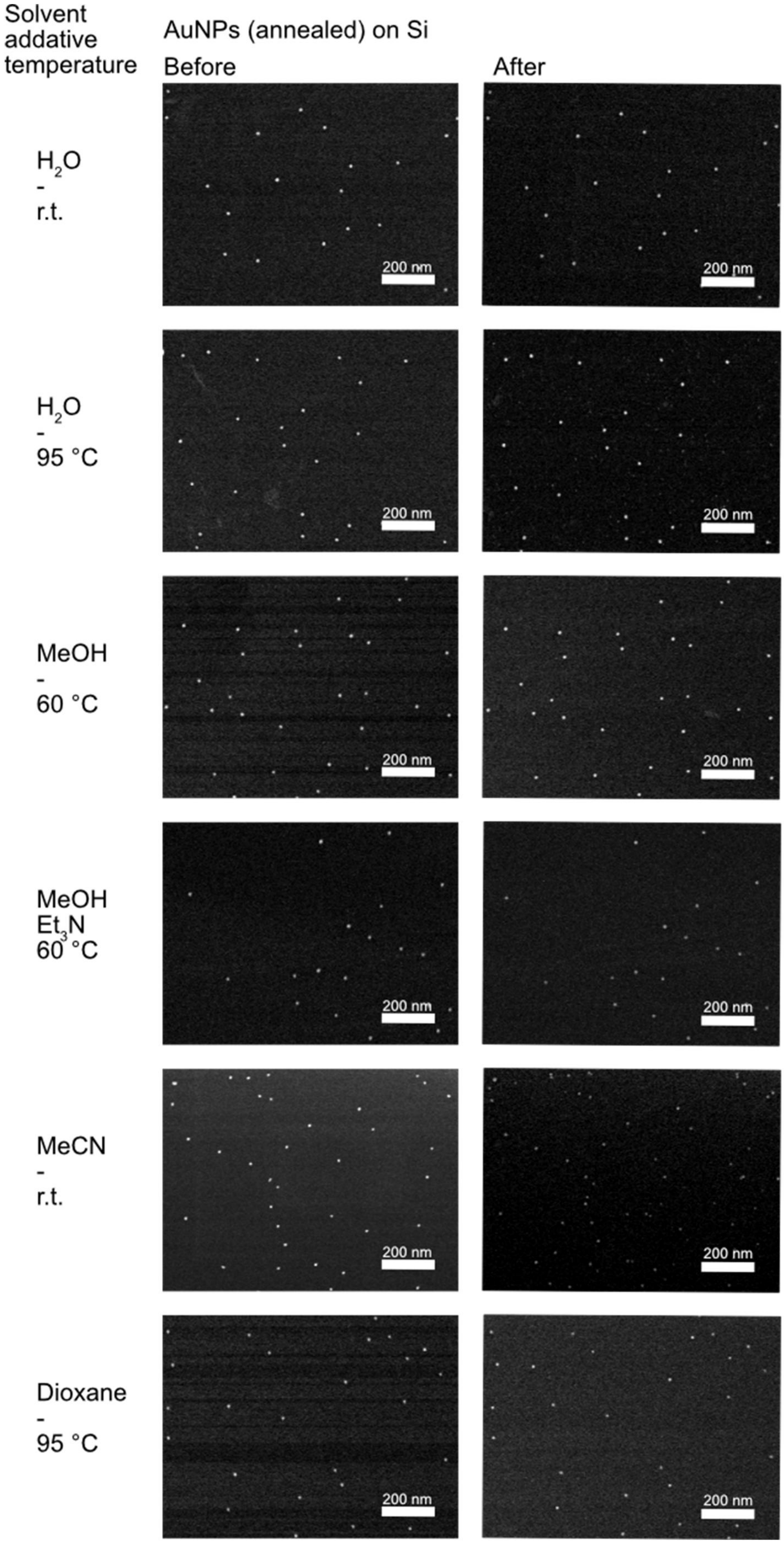

Solvent
addative
temperature
AuNPs (annealed) on Si
Before
After
H_2O
-
r.t.
H_2O
-
95 °C
MeOH
-
60 °C
MeOH
Et_3N
60 °C
MeCN
-
r.t.
Dioxane
-
95 °C
200 nm

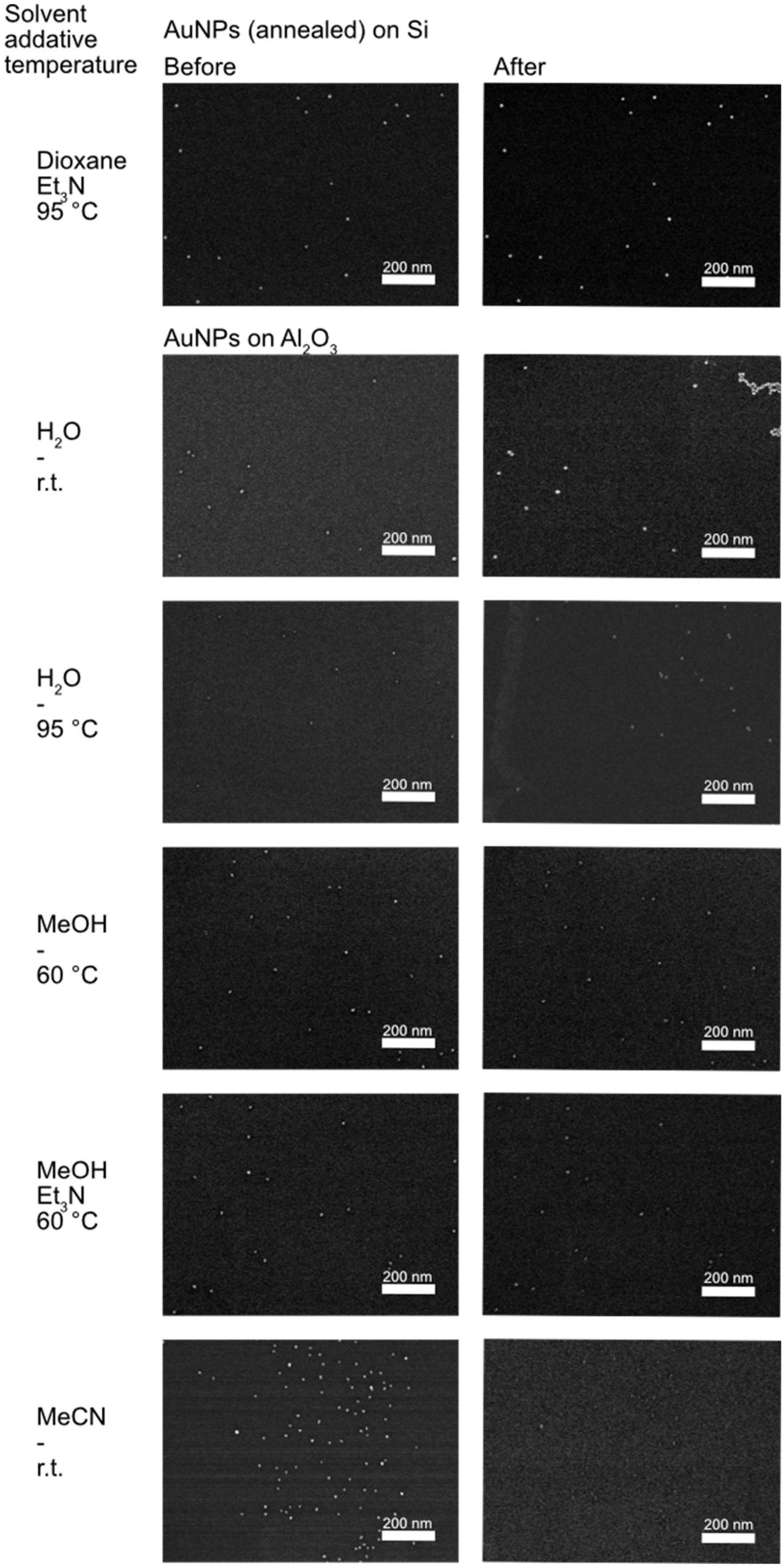
Solvent
addative
temperature
AuNPs (annealed) on Si
Before
After
Dioxane
Et_3N
95 °C
200 nm
AuNPs on Al_2O_3
H_2O
-
r.t.
H_2O
-
95 °C
MeOH
-
60 °C
MeOH
Et_3N
60 °C
MeCN
-
r.t.

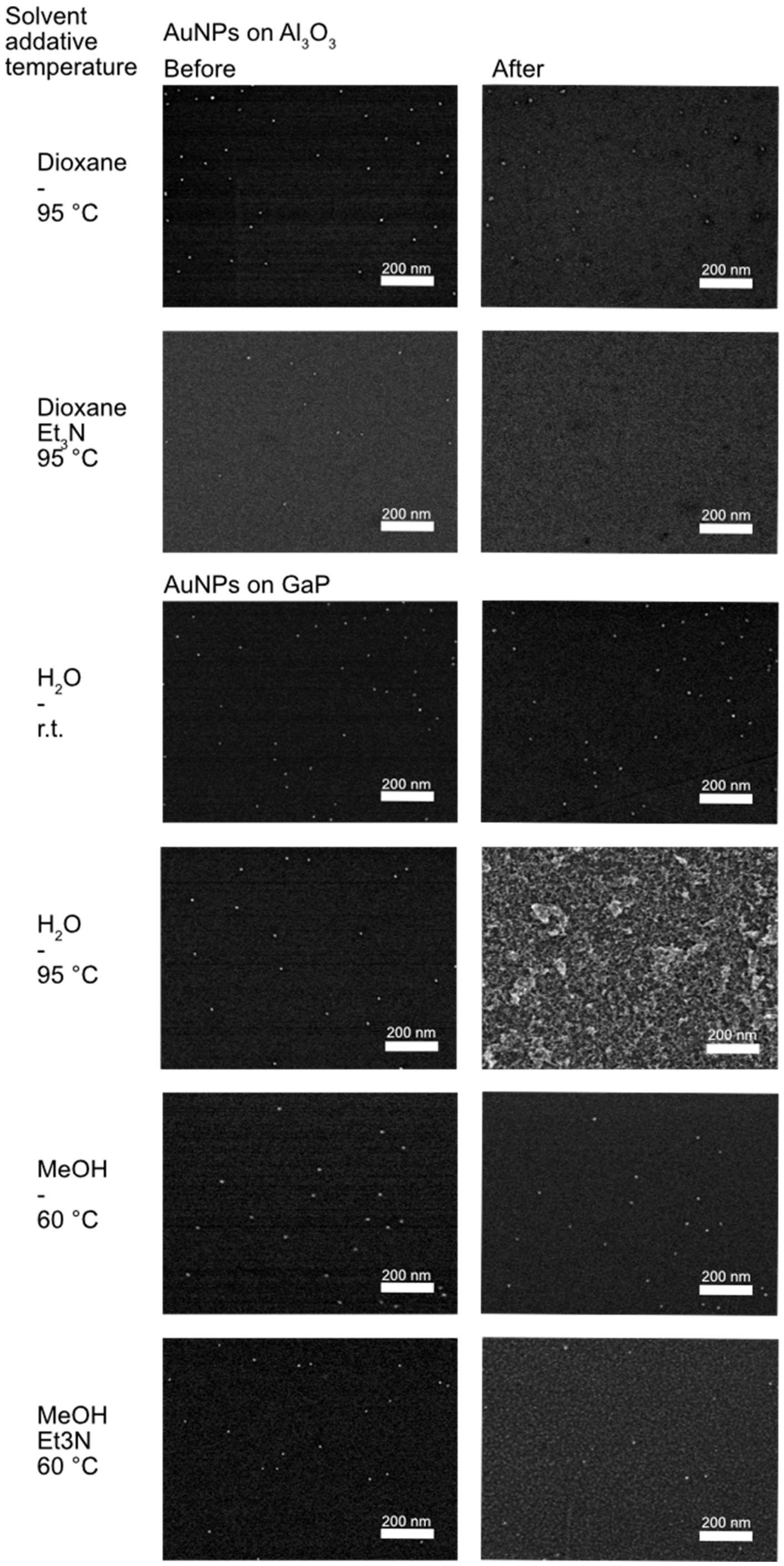
Solvent
addative
temperature
AuNPs on Al3O3
Before
After
Dioxane
-
95 °C
Dioxane
Et3N
95 °C
AuNPs on GaP
H2O
-
r.t.
H2O
-
95 °C
MeOH
-
60 °C
MeOH
Et3N
60 °C
200 nm

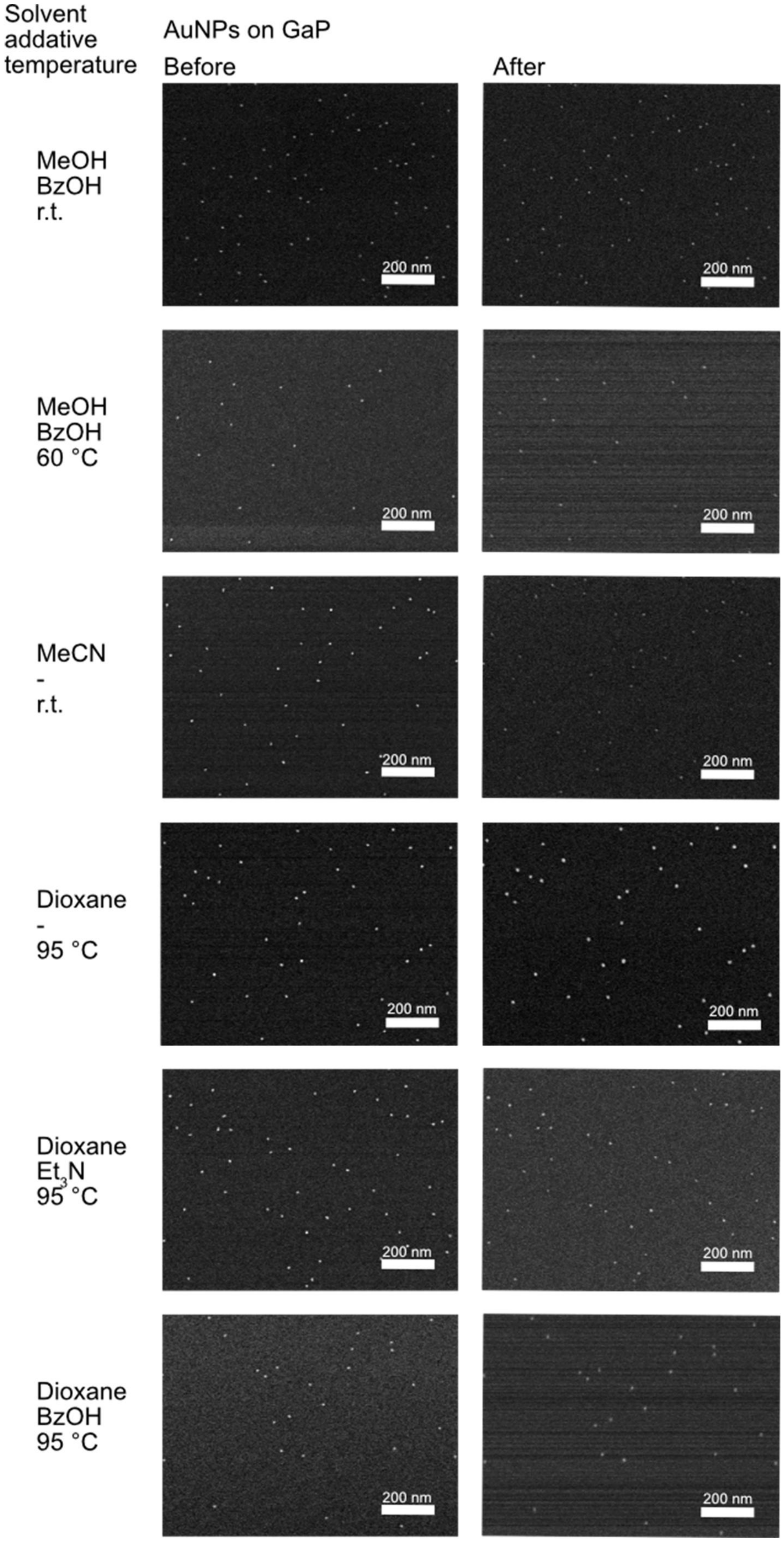
Solvent
addative
temperature
AuNPs on GaP
Before
After
MeOH
BzOH
r.t.
MeOH
BzOH
60 °C
MeCN
-
r.t.
Dioxane
-
95 °C
Dioxane
Et3N
95 °C
Dioxane
BzOH
95 °C
200 nm

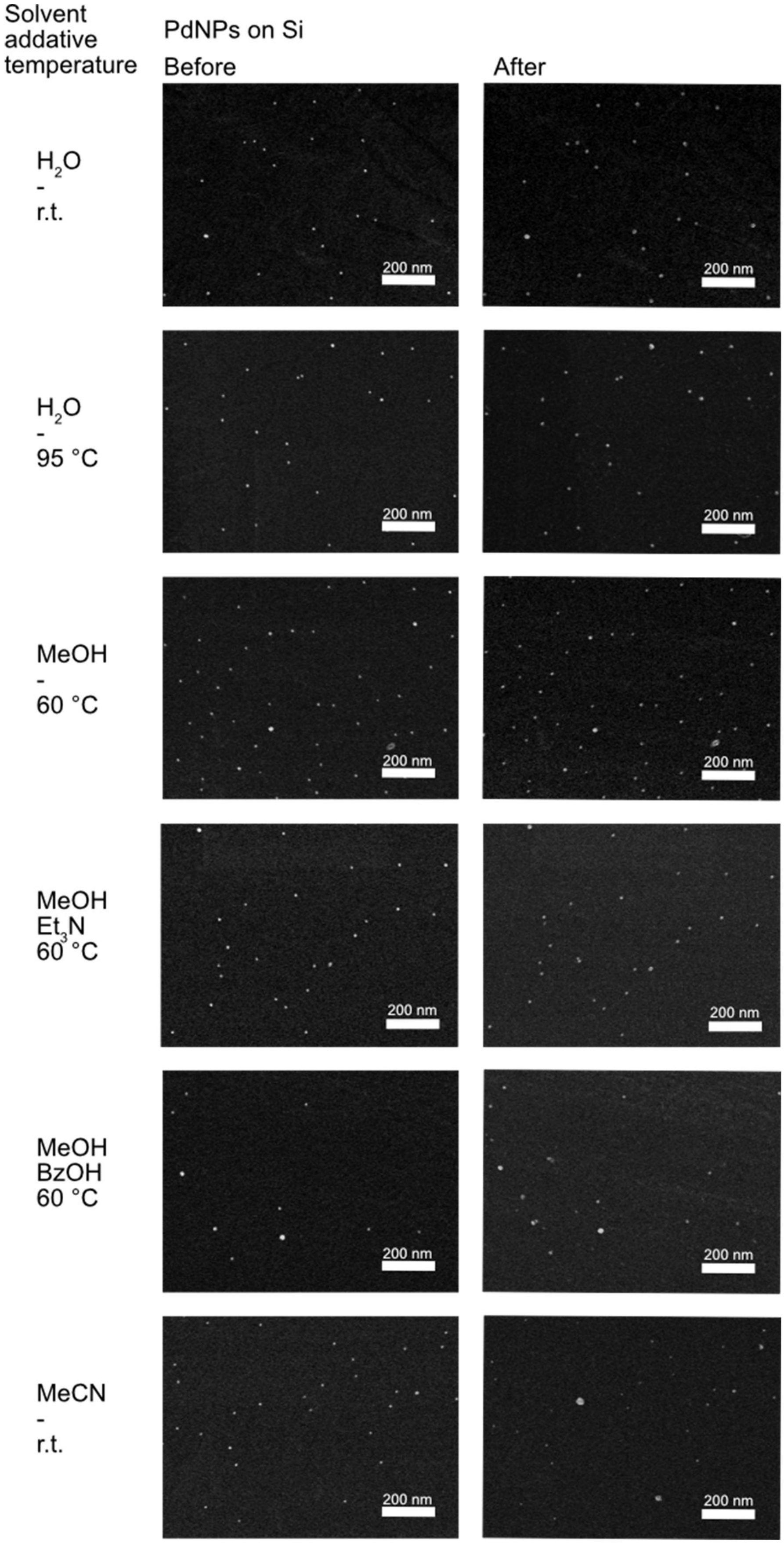
Solvent
addative
temperature
PdNPs on Si
Before
After
H2O
-
r.t.
H2O
-
95 °C
MeOH
-
60 °C
MeOH
Et3N
60 °C
MeOH
BzOH
60 °C
MeCN
-
r.t.
200 nm

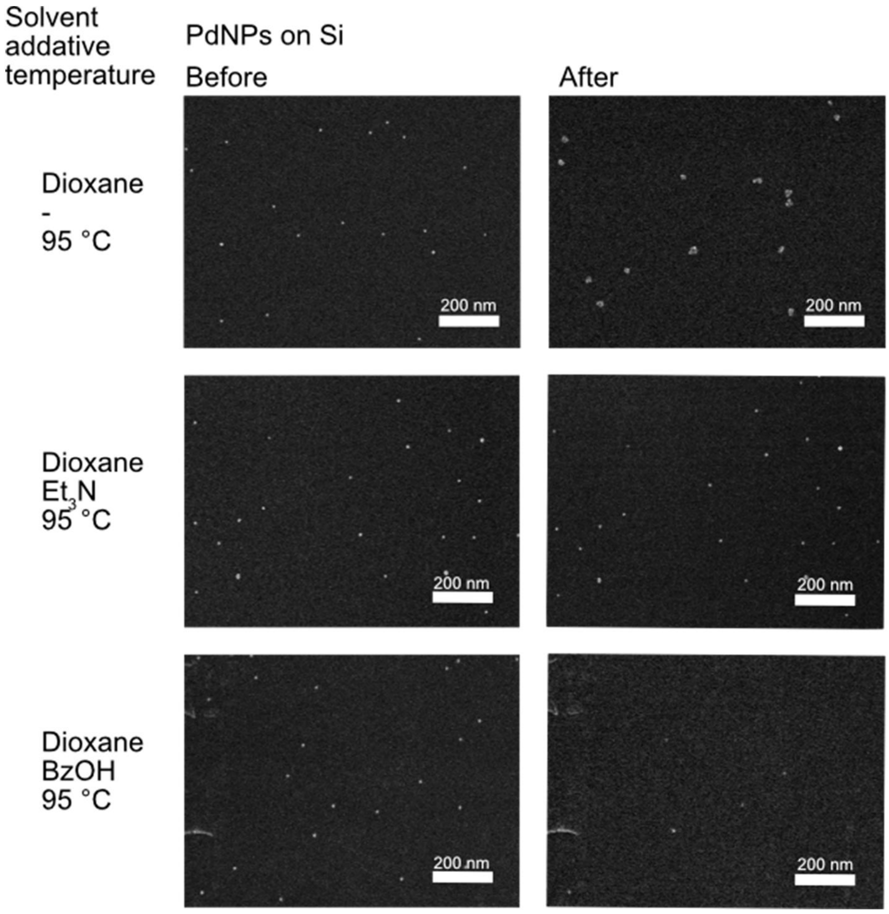

Figure S2. Representative SEM images acquired in the reference areas before and after treatment in solution. Note that these images only display a small part of the total studied area. Abbreviations: PBS=phosphate-buffered saline, Et_3N=triethylamine, MeCN=acetonitrile, BzOH=benzoic acid, DCM=dichloromethane, DCE=1,2-dichloroethane, MeOH=methanol, DMF=dimethylformamide.

C. Particle size distributions

In this section histograms of particle size distributions are presented. The size distributions were obtained from SEM images using the software ImageJ and their built-in macro "Analyze particles". The macro identifies and measures the area of any particles within a set area range, here set to 5 nm^2–infinity. The lower limit was set to avoid identifying noise as particles. From the measured area, the diameter of the particles were calculated, assuming spherical particles.

Figure S3 and S4 show the particle size distribution of the supported gold and palladium nanoparticles, respectively. Figure S5 include histograms of all the different supported nanoparticle chips before and

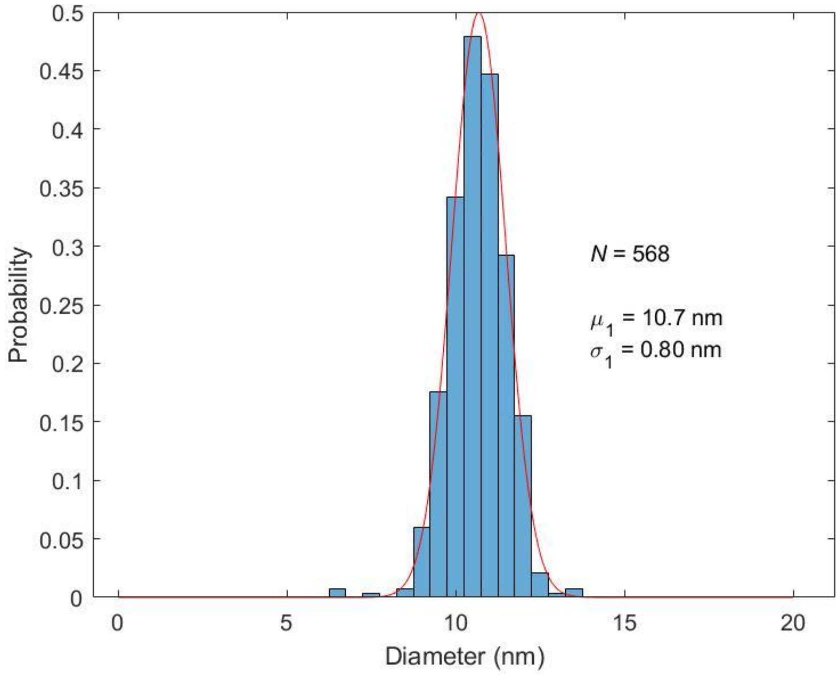

Figure S3. Size distribution of Au nanoparticles on Si with mean diameter of 10.7 nm. Based on 568 particles.

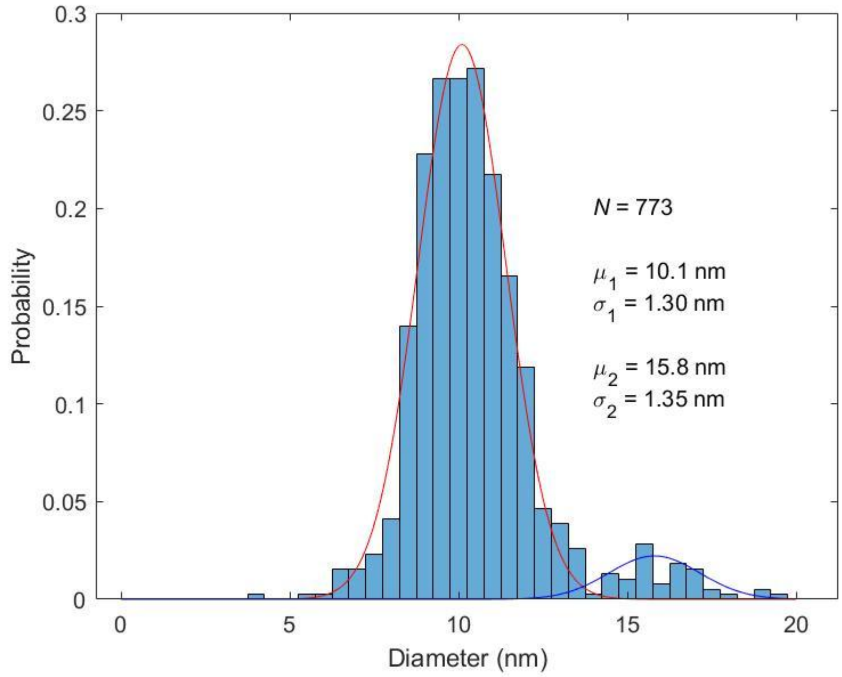

Figure S4. Size distribution of Pd nanoparticles on Si. The distribution fits two Gaussian distributions where 92% of the particles fall within the main distribution with a mean diameter of 10.1 nm. 8% of the particles follow a second distribution with a mean diameter of 15.8 nm. Their presence can be attributed to double-charged particles during the size selection. Based on 773 particles.

after treatment in the cases where etching was observed, showing a broadening of the nanoparticle size distribution after treatment.

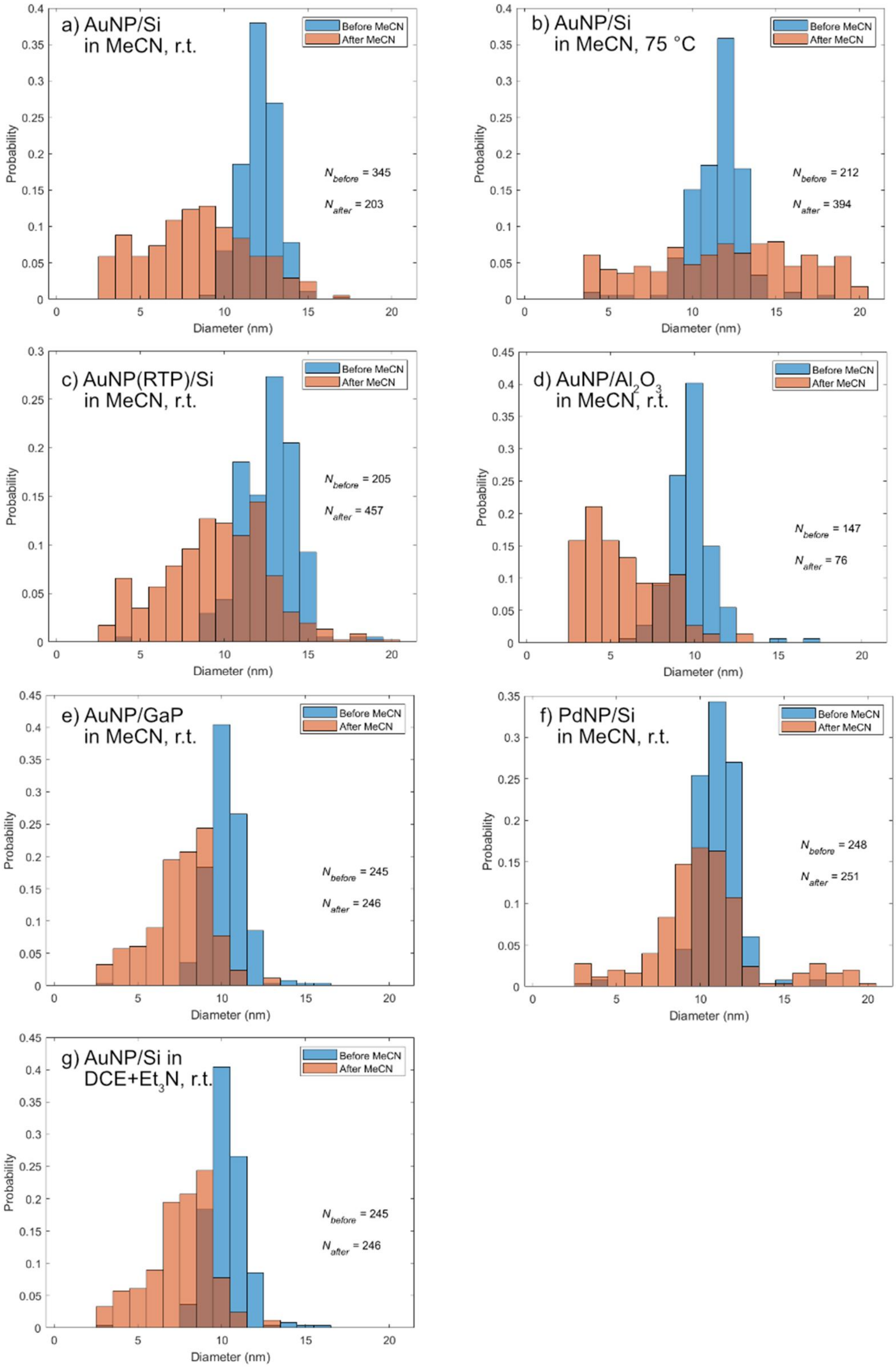

Figure S5. Size distribution of the different supported nanoparticle chips before and after treatment for 24 hours in the cases where etching of the nanoparticles was observed.

D. Data stability tests

Table S1. Summary of the experimental data of nanoparticle movement in the reference areas (IL-SEM) as well as particle density decrease and possible clustering outside the reference areas. The data is the average value from a minimum of two independent samples. The last column shows the concluded alternation type. The alternation type *Minor movement* and *Major movement* is based on the data shown, while the alternation type *Etched* particles and surface is based on the visual interpretation of the SEM images combined with histograms of the particle size distributions (Figure S5). Abbreviations: PBS=phosphate-buffered saline, Et_3N=triethylamine, MeCN=acetonitrile, BzOH=benzoic acid, DCM=dichloromethane, DCE=1,2-dichloroethane, MeOH=methanol, DMF=dimethylformamide.

Conditions			Support/NP info		Reference area (IL-SEM)		Outside reference area		
Solvent	Additive	Temp.	Support material	NP material	No. moved/removed particles	No. additional particles	Decreased particle density (%)	Linked/ clustered particles? (Y/N)	Comment
H_2O	-	r.t.	Si	Au	2	18	6	N	Minor movement
	-	95 °C	Si	Au	4	7	0	N	Minor movement
	-	r.t.	Al_2O_3	Au	14	67	59	N	Major movement
	-	95 °C	Al_2O_3	Au	29	10	10	N	Major movement
	-	r.t.	GaP	Au	4	19	15	Y	Minor movement
	-	95 °C	GaP	Au	-	-	-	-	Etched surface
	-	r.t.	Si	Au(RTP)	0	0	0	N	
	-	95 °C	Si	Au(RTP)	0	2	3	N	
	Et_3N	r.t.	Si	Au(RTP)	1	3	0	N	
	-	r.t.	Si	Pd	0	0	11	N	
PBS-buffer	-	37 °C	Si	Au	0	0	20	N	Residues
MeCN	-	r.t.	Si	Au	-	0	-	-	Etched particles
		75 °C	Si	Au	-	0	-	-	Etched particles
	-	r.t.	Al_2O_3	Au	-	-	100	-	Etched particles
	-	r.t.	GaP	Au	3	0	100	-	Etched particles
	-	r.t.	Si	Au(RTP)	1	100	0	Y	Etched particles

	-	r.t.	Si	Pd	1	14	0	Y	Etched particles
Toluene	-	r.t.	Si	Au	0	0	0	N	
	-	95 °C	Si	Au	0	0	0	N	
	Et_3N	r.t.	Si	Au	0	0	0	N	
	Et_3N	95 °C	Si	Au	1	0	6	N	
Dioxane	-	r.t.	Si	Au	0	0	0	N	
	-	95 °C	Si	Au	0	0	0	N	
	Et_3N	r.t.	Si	Au	0	0	0	N	
	Et_3N	95 °C	Si	Au	0	0	0	N	
	BzOH	r.t.	Si	Au	1	0	0	N	
	BzOH	95 °C	Si	Au	2	2	5	N	
	-	95 °C	Al_2O_3	Au	52	0	15	Y	Major movement
	Et_3N	95 °C	Al_2O_3	Au	33	0	62	N	Major movement
	-	95 °C	GaP	Au	0	0	2	N	
	Et_3N	95 °C	GaP	Au	0	0	18	N	
	BzOH	95 °C	GaP	Au	1	1	4	N	
	-	95 °C	Si	Au(RTP)	0	0	0	N	
	Et_3N	95 °C	Si	Au(RTP)	0	0	0	N	
	-	95 °C	Si	Pd	1	1	52	Y	Major movement
	Et_3N	95 °C	Si	Pd	0	1	6	N	
	BzOH	95 °C	Si	Pd	25	25	87	Y	Major movement
DCM	-	r.t.	Si	Au	0	0	0	N	
	-	35 °C	Si	Au	0	0	0	N	
	Et_3N	r.t.	Si	Au	0	1	0	N	
	Et_3N	35 °C	Si	Au	0	0	4	N	
DCE	-	r.t.	Si	Au	0	0	0	N	
	-	75 °C	Si	Au	0	0	0	N	
	Et_3N	r.t.	Si	Au	0	0	3	N	
	Et_3N	75 °C	Si	Au	74	0	80	N	Major movement
MeOH	-	r.t.	Si	Au	0	0	6	N	
	-	60 °C	Si	Au	0	0	0	N	

	Et_3N	r.t.	Si	Au	1	38	55	Y	Major movement
	Et_3N	60 °C	Si	Au	0	3	25	Y	Minor movement
	BzOH	r.t.	Si	Au	0	0	0	N	
	BzOH	60 °C	Si	Au	1	29	3	Y	Minor movement
	-	60 °C	Al_2O_3	Au	0	0	0	N	
	Et_3N	60 °C	Al_2O_3	Au	0	0	3	N	
	-	60 °C	GaP	Au	1	0	0	N	
	Et_3N	60 °C	GaP	Au	20	0	61	N	
	BzOH	r.t.	GaP	Au	0	0	0	N	
	BzOH	60 °C	GaP	Au	0	0	10	N	
	-	60 °C	Si	Au(RTP)	0	0	4	N	
	Et_3N	60 °C	Si	Au(RTP)	0	0	0	N	
	-	60 °C	Si	Pd	0	0	0	N	
	Et_3N	60 °C	Si	Pd	1	0	13	Y	Minor movement
	BzOH	60 °C	Si	Pd	1	0	7	Y	Minor movement
DMF	-	r.t.	Si	Au	0	0	0	N	
	-	95 °C	Si	Au	1	0	0	N	
	Et_3N	r.t.	Si	Au	0	0	53	N	
	Et_3N	60 °C	Si	Au	0	0	30	N	
	Et_3N	95 °C	Si	Au	100	0	100	-	
Ethylene glycol	-	r.t.	Si	Au	0	0	0	N	
	-	95 °C	Si	Au	4	0	0	Y	Minor movement
	Et_3N	r.t.	Si	Au	0	0	7	N	
	Et_3N	95 °C	Si	Au	0	0	79	Y	Major movement
